# The role of electrical stimulation for rehabilitation and regeneration after spinal cord injury

**DOI:** 10.1186/s10195-021-00623-6

**Published:** 2022-01-06

**Authors:** Brian A. Karamian, Nicholas Siegel, Blake Nourie, Mijail D. Serruya, Robert F. Heary, James S. Harrop, Alexander R. Vaccaro

**Affiliations:** 1 Rothman Orthopaedic Institute at Thomas Jefferson University, 925 Chestnut St, 5th Floor, Philadelphia, PA 19107 USA; 2 Thomas Jefferson University, Philadelphia, PA 19107 USA; 3 Department of Neurological Surgery, Hackensack Meridian School of Medicine, Nutley, NJ 07110 USA

**Keywords:** Spinal cord injury, Electrical stimulation, Functional electrical stimulation, Neurorehabilitation, Neuroprosthesis, Neuroplasticity

## Abstract

Electrical stimulation is used to elicit muscle contraction and can be utilized for neurorehabilitation following spinal cord injury when paired with voluntary motor training. This technology is now an important therapeutic intervention that results in improvement in motor function in patients with spinal cord injuries. The purpose of this review is to summarize the various forms of electrical stimulation technology that exist and their applications. Furthermore, this paper addresses the potential future of the technology.

## Epidemiology

Globally, approximately 250,000 to 500,000 new spinal cord injury (SCI) cases occur every year [1]. Blunt force trauma is primarily responsible for SCI, with motor vehicle crashes serving as the leading cause of injury (38.2%), followed by falls (32.3%) [2]. Medical expenses over US $3 billion are spent annually on managing SCI, and individual costs can range from US $380,000 to US $1,160,000 in the first year alone, and between US $46,000 and US $202,000 for each subsequent year [2].

## Sequelae

Neurologic injury of the spinal cord affects nearly every physiologic system, and patients can present with a multitude of symptoms that drastically influence their function and quality of life. The SCI level determines which systems are affected and has a significant impact on the potential rehabilitation and final functional status of the patient.

### Musculoskeletal system

While C1–C4 SCI typically results in tetraplegia, lower cervical (C5–C8) SCI can spare varying degrees of upper extremity function. The C5 nerve root primarily innervates the deltoid muscle to perform shoulder abduction, but is also responsible elbow flexion. Accordingly, C5 complete SCI (ASIA A) results in complete dependence for transfers and assistance for activities of daily living. The C6 nerve root controls wrist extension and biceps flexion, the C7 nerve root controls elbow extension and wrist flexion, and the C8 nerve roots controls finger flexion. SCI below C6 results in relatively greater independence, with patients able to achieve transfers either with the assistance of a transfer board (C6) or independently (C7/C8). These patients require less assistance and fewer adaptive aids for activities of daily living. Any complete level thoracic SCI results in paraplegia, however, SCI distal to L2 level may spare varying lower extremity function.

Damage to descending spinal cord tracks results in hyperexcitability and spasticity [3, 4]. Spasticity is a velocity-dependent increase in muscle tone due to a hyperexcitable stretch reflex [5]. Spasticity may potentially have beneficial effects by promoting venous return, decreasing the incidence of orthostatic hypertension and deep venous thrombosis, increasing stability, and facilitating activities such as transfers [3, 6, 7]. However, these must be weighed against the negative effects that include contractures, gait disturbances, decreased mobility, and pain [3, 8, 9].

Due to mobility limitations, paraplegia or tetraplegia patients do not load their spine or limbs, disturbing bone homeostasis as a result of mechanical unloading. Cessation of weight bearing in these patients leads to increased bone resorption and suppressed bone formation. The resulting osteoporosis is typically isolated to the long bones below the level of injury, increasing the risk of fragility fractures [10–13].

### Integumentary system

One of the most common adverse events following an SCI is pressure ulcers due to insensate regions. Ulceration occurs due to persistent pressure over bony prominences as a result of immobility, poor nutrition, and changes in skin physiology including deficient vascular reactions to catecholamine signaling and decreased fibroblast activity. These changes delay the natural wound healing capabilities below the level of the injury, resulting in ulceration [14, 15]. The annual incidence of pressure ulcers in SCI patients ranges from 20% to 31%, with the resulting increased healthcare utilization approximately quadrupling annual costs compared with SCI patient without ulcers [16, 17].

### Cardiopulmonary system

SCI in the cervical or high thoracic regions can disrupt respiratory muscle function, ranging from exercise intolerance to complete respiratory failure requiring mechanical ventilation assistance [18]. In patients with prolonged ventilation, tracheostomy may be required. Poor respiratory muscle recruitment in combination with inhibited reflexes results in impaired cough, bronchospasm, and increased secretions, predisposing SCI patients to pneumonia, atelectasis, and exacerbation of respiratory failure [14, 19].

SCI patients also have increased risk of ischemic heart disease because of the increased prevalence of coronary artery disease (CAD) and hypertension after SCI [20]. The prevalence of symptomatic cardiovascular disease ranges from 30% to 50% compared with 5–10% in matched able-bodied populations [21, 22]. CAD risk factors, including hyperlipidemia, diabetes, and obesity, that exist within the SCI population have primarily been attributed to the sedentary nature of SCI patients [23–27].

### Sympathetic nervous system

SCI proximal to T6 level may result in autonomic dysreflexia, affecting autonomic responses to demands on vascular tone and heart rate, with greater severity of dysregulation associated with higher levels of injury [28, 29]. Autonomic dysreflexia results in sympathetic over activity causing hypertension that increases the risk of stroke, pain, and hemodynamic instability. Parasympathetic compensation, including bradycardia and vasodilation, occurs only above the level of the injury, resulting in sweating, chills, headache, and flushing [30, 31]. Dysreflexia is often initiated by noxious stimuli below the level of the spinal cord injury, including cutaneous or visceral etiologies, but is most often triggered by a urologic source such as urinary tract infection or bladder distention [32]. Injuries below T6 do not typically result in autonomic dysreflexia due to the intact splanchnic innervation [33].

### Urinary system

SCI can disrupt both storage and emptying of the bladder. The majority of bladder dysfunction results from detrusor overactivity causing urge incontinence. Patients can also have detrusor sphincter dyssynergia, where the bladder contracts against a hyperactive closed sphincter leading to vesicoureteral reflux [34–38]. Detrusor areflexia has also been noted in SCI patients with involvement of lower motor neurons resulting in chronic urinary retention with incomplete emptying and overflow incompetence [38]. Due to these conditions, many patients require intermittent catheterization or indwelling catheters that increase the risk of developing urinary tract infections (UTIs) [38–41]. Patients with SCI are also noted to have an increased incidence of nephrolithiasis secondary to immobilization hypercalciuria, which may also predispose patients to UTIs [14, 38, 42–45].

### Reproductive system

In addition to urologic impairments, SCI often results in sexual dysfunction. The incidence of impotence in men after SCI is approximately 75%, where the level of the injury dictates the type of sexual dysfunction. If there is a lower motor neuron lesion at the level of the sacral roots, parasympathetic innervation will be interrupted and reflexogenic erections are impacted (i.e., tactile stimulation resulting in an erection). Alternatively, psychogenic erections are mediated through sympathetic pathways originating from T10–T12. As such, psychologically mediated erections are possible in patients with injuries caudal to T12 [38]. There is a paucity of literature on sexual dysfunction in women with SCI, but an impaired ability to achieve orgasm after SCI has been described [38].

### Classifications of neurologic injury

The Frankel scale was introduced in 1969 as a 5-point scale to grade SCI [46]. Patients are classified as complete (grade A), sensory only (grade B), motor useless (grade C), motor useful (grade D), or no neurological deficit/complete recovery (grade E). Continued use of this scale was limited by its subjective nature in judging “usefulness” of any remaining motor movements and its failure to account for the level of injury [46]. The American Spinal Injury Association published the International Standards for Neurological Classification of Spinal Injury in 1982 [47]. This classification has evolved into the current American Spinal Injury Association Impairment Scale (AIS) [48]. In contrast to the Frankel system, the AIS improves reproducibility via standardized testing of myotomes and dermatomes to identify the level of injury [49, 50]. Additionally, the AIS differentiates between complete and incomplete injuries.

The AIS is now the international standard for evaluation and classification of patients with SCI [50]. The scale grades A–E: Patients with Grade A have complete spinal cord injuries and as such, have no motor or sensory function (including sacral roots) distal to the level of injury. Patients with Grade B have some sensory function, but no motor function below the level of injury. Grade C injuries consist of a motor strength less than 3/5 in more than half of the major muscle groups below the level of injury, while Grade D injuries have a motor grade 3/5 or greater. Patients with Grade E have full motor and sensory function after sustaining a SCI [48].

## Electrical simulation

Spinal cord injury is a discontinuity syndrome that disrupts efferent and afferent pathways, including the descending motor fibers from the motor cortex to the spinal motor neurons and the ascending somatosensory fibers from the peripheral nervous system through the spinal cord and to the brain [51]. In other regions of the body, conduction blockage is resolved with regrowth, regeneration, and sometimes functional reconnectivity of axons to the end organ resulting in functional recovery. However, the central nervous system has impaired ability to restore neural circuits across a lesion. Accordingly, scientific innovations have been created to bypass the area of injury and reconnect end organ function.

The use of electrical stimulation after spinal cord injuries has been utilized for over half a century [52]. One of the earliest uses of electrical stimulation in the 1960s utilized electrical current to stimulate the peroneal nerve to initiate muscle function and correct foot drop in the setting of stroke-related hemiplegia [53]. Since then, various types of electrical stimulation have been developed and employed to conserve the function of the aforementioned physiologic systems (Table 1).

**Table 1:** Summarized literature references by topic

Title	Authors
Transcutaneous electrical neural stimulation	
Relief of hemiparetic spasticity by TENS is associated with improvement in reflex and voluntary motor functions	Levin et al. [60]
Patterned sensory stimulation induces plasticity in reciprocal Ia inhibition in humans	Perez et al. [61]
Electrical stimulation in treating spasticity resulting from spinal cord injury	Bajd et al. [62]
Neuromuscular electrical stimulation	
Electrical treatment of spasticity. Reflex tonic activity in hemiplegic patients and selected specific electrostimulation	Alfieri [64]
Two theories of muscle strength augmentation using percutaneous electrical stimulation	Delitto et al. [65]
Neuromuscular electrical stimulation-induced resistance training after SCI: a review of the Dudley protocol	Bickel et al. [66]
Neuromuscular electrical stimulation in neurorehabilitation	Sheffler et al. [67]
Electrical stimulation of wrist extensors in poststroke hemiplegia	Powell et al. [68]
Functional electrical stimulation	
Functional electrical stimulation therapy for restoration of motor function after spinal cord injury and stroke: a review	Marquez-Chin et al. [69]
Functional electrical stimulation in spinal cord injury: from theory to practice	Martin et al. [70]
Functional electrical stimulation and spinal cord injury	Ho et al. [71]
Functional electrical stimulation post-spinal cord injury improves locomotion and increases afferent input into the central nervous system in rats	Beaumont et al. [72]
Functional electrical stimulation for neuromuscular applications	Peckham et al. [73]
Surface-stimulation technology for grasping and walking neuroprostheses: improving quality of life in stroke/spinal cord injury subjects with rapid prototyping and portable FES systems	Popovic et al. [74]
An update on functional electrical stimulation after spinal cord injury	Gorman [75]
Paradigms of lower extremity electrical stimulation training after spinal cord injury	Gorgey et al. [76]
Transcutaneous functional electrical stimulation for grasping in subjects with cervical spinal cord injury	Mangold et al. [77]
Influence of different rehabilitation therapy models on patient outcomes: hand function therapy in individuals with incomplete SCI	Kapadia et al. [78]
Functional electrical stimulation therapy of voluntary grasping versus only conventional rehabilitation for patients with subacute incomplete tetraplegia: a randomized clinical trial	Popovic et al. [79]
A noninvasive neuroprosthesis augments hand grasp force in individuals with cervical spinal cord injury: the functional and therapeutic effects	Thorsen et al. [80]
A clinically meaningful training effect in walking speed using functional electrical stimulation for motor-incomplete spinal cord injury	Street et al. [81]
Implanted functional electrical stimulation: an alternative for standing and walking in pediatric spinal cord injury	Johnston et al. [82]
Restoration of gait by functional electrical stimulation in paraplegic patients: a modified programme of treatment	Maležič et al. [83]
A randomized trial of functional electrical stimulation for walking in incomplete spinal cord injury: effects on walking competency	Kapadia et al. [84]
Therapeutic effects of functional electrical stimulation on gait, motor recovery, and motor cortex in stroke survivors	Shendkar et al. [85]
The effectiveness of functional electrical stimulation for the treatment of shoulder subluxation and shoulder pain in hemiplegic patients: a randomized controlled trial	Koyuncu et al. [86]
Role of electrical stimulation for rehabilitation and regeneration after spinal cord injury: an overview	Hamid et al. [51]
Functional electrical stimulation of dorsiflexor muscle: effects on dorsiflexor strength, plantarflexor spasticity, and motor recovery in stroke patients	Sabut et al. [87]
The efficacy of electrical stimulation in reducing the post-stroke spasticity: a randomized controlled study	Sahin et al. [88]
Functional electric stimulation-assisted rowing: increasing cardiovascular fitness through functional electric stimulation rowing training in persons with spinal cord injury	Wheeler et al. [89]
Efficacy of electrical stimulation for spinal fusion: a systematic review and meta-analysis of randomized controlled trials	Akhter et al. [90]
Functional electrical stimulation therapies after spinal cord injury	Gater et al. [91]
An externally powered, multichannel, implantable stimulator-telemeter for control of paralyzed muscle	Smith et al. [92]
Implanted functional neuromuscular stimulation systems for individuals with cervical spinal cord injuries: clinical case reports	Triolo et al. [93]
Efficacy of an implanted neuroprosthesis for restoring hand grasp in tetraplegia: a multicenter study	Peckham et al. [94]
Factors influencing body composition in persons with spinal cord injury: a cross-sectional study	Spungen et al. [96]
The effects of trunk stimulation on bimanual seated workspace	Kukke et al. [97]
Effects of stimulating hip and trunk muscles on seated stability, posture, and reach after spinal cord injury	Triolo et al. [98]
The effects of combined trunk and gluteal neuromuscular electrical stimulation on posture and tissue health in spinal cord injury	Wu et al. [99]
Long-term performance and user satisfaction with implanted neuroprostheses for upright mobility after paraplegia: 2- to 14-year follow-up	Triolo et al. [101]
An approach for the cooperative control of FES with a powered exoskeleton during level walking for persons with paraplegia	Ha et al. [102]
Functional neuromuscular stimulator for short-distance ambulation by certain thoracic-level spinal-cord-injured paraplegics	Graupe et al. [103]
Phrenic nerve pacing	
Diaphragm pacing for respiratory insufficiency	Chervin et al. [105]
Diaphragm pacing by electrical stimulation of the phrenic nerve	Glenn et al. [106]
Multicenter review of diaphragm pacing in spinal cord injury: successful not only in weaning from ventilators but also in bridging to independent respiration	Posluszny et al. [107]
Successful reinnervation of the diaphragm after intercostal to phrenic nerve neurotization in patients with high spinal cord injury	Nandra et al. [108]
Spinal cord stimulation	
Restoration of sensorimotor functions after spinal cord injury	Dietz et al. [110]
Transcutaneous spinal cord stimulation restores hand and arm function after spinal cord injury	Inanici et al. [111]
Transcutaneous electrical spinal stimulation promotes long-term recovery of upper extremity function in chronic tetraplegia	Inanici et al. [112]
Transcutaneous electrical spinal-cord stimulation in humans	Gerasimenko et al. [113]
Non-invasive activation of cervical spinal networks after severe paralysis	Gad et al. [114]
Weight bearing over-ground stepping in an exoskeleton with non-invasive spinal cord neuromodulation after motor complete paraplegia	Gad et al. [115]
An autonomic neuroprosthesis: noninvasive electrical spinal cord stimulation restores autonomic cardiovascular function in individuals with spinal cord injury	Phillips et al. [116]
Transcutaneous spinal cord stimulation and motor rehabilitation in spinal cord injury: a systematic review	Megia Garcia et al. [117]
Configuration of electrical spinal cord stimulation through real-time processing of gait kinematics	Capogrosso et al. [119]
Targeted neurotechnology restores walking in humans with spinal cord injury	Wagner et al. [120]
Spatiotemporal neuromodulation therapies engaging muscle synergies improve motor control after spinal cord injury	Wenger et al. [121]
Cardiovascular autonomic dysfunction in spinal cord injury: epidemiology, diagnosis, and management	Wecht et al. [124]
Autonomic neuromodulation	
New approaches for treating atrial fibrillation: focus on autonomic modulation	Sohinki et al. [125]
Neuromodulation for the treatment of heart rhythm disorders	Waldron et al. [126]
Low-level vagus nerve stimulation attenuates myocardial ischemic reperfusion injury by antioxidative stress and antiapoptosis reactions in canines	Chen et al. [127]
Closed-loop neuromodulation restores network connectivity and motor control after spinal cord injury	Ganzer et al. [128]
Acute cardiovascular responses to vagus nerve stimulation after experimental spinal cord injury	Sachdeva et al. [129]
Vagus nerve stimulation paired with rehabilitative training enhances motor recovery after bilateral spinal cord injury to cervical forelimb motor pools	Darrow et al. [130]
Cross-modal plasticity revealed by electrotactile stimulation of the tongue in the congenitally blind	Ptito et al. [131]
Sustained cortical and subcortical neuromodulation induced by electrical tongue stimulation	Wildenberg et al. [132]
High-resolution fMRI detects neuromodulation of individual brainstem nuclei by electrical tongue stimulation in balance-impaired individuals	Wildenberg et al. [133]
Electrical tongue stimulation normalizes activity within the motion-sensitive brain network in balance-impaired subjects as revealed by group independent component analysis	Wildenberg et al. [134]
Altered connectivity of the balance processing network after tongue stimulation in balance-impaired individuals	Wildenberg et al. [135]
Feasibility of sensory tongue stimulation combined with task-specific therapy in people with spinal cord injury: a case study	Chisholm et al. [136]
Cranial nerve non-invasive neuromodulation improves gait and balance in stroke survivors: a pilot randomised controlled trial	Galea et al. [137]
A prospective, multicenter study to assess the safety and efficacy of translingual neurostimulation plus physical therapy for the treatment of a chronic balance deficit due to mild‐to‐moderate traumatic brain injury	Ptito et al. [138]
Sacral nerve stimulation	
Design and implementation of low-power neuromodulation S/W based on MSP430	Hong et al. [139]
Electrical stimulation of sacral dermatomes can suppress aberrant urethral reflexes in felines with chronic spinal cord injury	McCoin et al. [140]
Neuromodulation for restoration of urinary and bowel control	Raina [141]
Early sacral neuromodulation prevents urinary incontinence after complete spinal cord injury	Sievert et al. [142]
Bladder neuromodulation in acute spinal cord injury via transcutaneous tibial nerve stimulation: cystometrogram and autonomic nervous system evidence from a randomized control pilot trial	Stampas et al. [143]
Lower urinary tract dysfunction in the neurological patient: clinical assessment and management	Panicker et al. [144]
Neuromodulation by surface electrical stimulation of peripheral nerves for reduction of detrusor overactivity in patients with spinal cord injury: a pilot study	Ojha et al. [145]
Galvanic vestibular stimulation	
Vestibulospinal responses in motor incomplete spinal cord injury	Liechti et al. [146]
Impaired scaling of responses to vestibular stimulation in incomplete SCI	Wydenkeller et al. [147]
Does galvanic vestibular stimulation decrease spasticity in clinically complete spinal cord injury?	Čobeljić et al. [148]
Transcranial direct current stimulation	
Evidence-based guidelines on the therapeutic use of transcranial direct current stimulation (tDCS)	Lefaucheur et al. [149]
Cortical vs. afferent stimulation as an adjunct to functional task practice training: a randomized, comparative pilot study in people with cervical spinal cord injury	Gomes-Osman et al. [150]
Improved grasp function with transcranial direct current stimulation in chronic spinal cord injury	Cortes et al. [151]
Effectiveness of anodal transcranial direct current stimulation to improve muscle strength and motor functionality after incomplete spinal cord injury: a systematic review and meta-analysis	de Araújo et al. [152]
Transcranial direct current stimulation is not effective in the motor strength and gait recovery following motor incomplete spinal cord injury during Lokomat® gait training	Kumru et al. [153]
Low-frequency rectangular pulse is superior to middle frequency alternating current stimulation in cycling of people with spinal cord injury	Szecsi et al. [155]
Oscillating field stimulation for complete spinal cord injury in humans: a phase 1 trial	Shapiro et al. [156]
Oscillating field stimulation promotes spinal cord remyelination by inducing differentiation of oligodendrocyte precursor cells after spinal cord injury	Zhang et al. [157]
Epidural oscillating field stimulation as an effective therapeutic approach in combination therapy for spinal cord injury	Bacova et al. [158]

Complete SCI prevents any signal from descending below the level of the injury due to incomplete circuitry. Even in completely injured patients, some circuits are spared, although these circuits are often not sufficient to establish an adequate level of excitability to stimulate motor neurons caudal to the injury. Electrical stimulation is believed to work by inducing neuroplastic changes at synapses within the spinal cord. Neuroplasticity is the process in which axons and synapses reorganize and adapt to their cellular environment.

After SCI, axon growth can include collateral sprouting of spared and injured axons, synaptic remodeling, and axon regeneration, albeit to a lesser extent than that which occurs outside the central nervous system [54]. Axonal sprouting and synaptic remodeling result in circuit reorganization, while axonal regeneration involves the regrowth of transected axons. Electrical stimulation induces neuroplasticity by increasing the baseline level of spinal excitability such that low levels of input result in voluntary motor function [55]. It has been hypothesized that the combination of electrical stimulation with voluntary motor commands is necessary to induce neuroplastic changes. When descending signals from the brain reach the corticospinal anterior horn synapse at the same time as antidromic signals traveling up the peripherally stimulated nerve by electrical stimulation, the synapse is strengthened and increases the probability of subsequent firing in a Hebbian-type learning effect, which postulates that an increase in synaptic efficiency arises from repeated stimulation [56]. This synaptic plasticity likely involves descending motor axons, proprioceptive afferents, motor neurons, and interneurons. By using electrical stimulation paired with voluntary motor training, the elicited neuroplasticity results in improvements in motor function.

### Transcutaneous electrical neural stimulation

Transcutaneous electrical neural stimulation (TENS) is a surface applied neuromodulation system that has been utilized in the treatment of various types of chronic pain, including noninvasive neuropathic pain relief through stimulation of sensory A-beta fibers and blocking of pain signals transmitted via A-delta and C-nociceptive fibers [57–59]. TENS is also used in the management of spasticity through a mechanism of neuroplasticity or modulation of inhibitory circuits [60–62]. TENS has been shown to enhance vibratory inhibition of the H reflex, the electrical equivalent of the monosynaptic stretch reflex, which has been attributed to presynaptic inhibition [60]. TENS treatment for spasticity enhances presynaptic inhibition, which is intrinsically suppressed in SCI patients. Furthermore, TENS resembling sensory feedback has been shown to induce short-term neuroplasticity by increasing the strength of reciprocal Ia inhibition between ankle flexor and extensor muscles [61].

### Neuromuscular electrical stimulation

Neuromuscular electrical stimulation (NMES) is electricity applied across the surface of the skin, and involves direct stimulation of targeted nerves to contract paralyzed muscles and increase muscle strength. NMES is thought to improve spasticity via disynaptic reciprocal inhibition in which the activation of one muscle produces an inhibition of the opposing muscle group [63, 64]. NMES is used to reverse muscle mass loss and improve functional movement similar to traditional muscle exercise [65, 66]. Furthermore, NMES is used in conjunction with repetitive movement therapy to facilitate motor relearning [67]. For example, NMES combined with standard rehabilitation has been shown to increase recovery of wrist extension over standard care in hemiplegic patients [68]. These therapeutic applications may lead to an effect that enhances but does not directly provide function. When NMES is used to directly accomplish functional tasks, it is called functional electrical stimulation (FES).

### Functional electrical stimulation

Functional electrical stimulation (FES) is a subtype of NMES that involves applying electrical stimuli to paralyzed nerves or muscles to induce muscular contraction in order to complete a functional task [69]. Conventional FES has been used in neurorehabilitation for tasks such as rowing or cycling [70, 71]. FES in neurorehabilitation is thought to support the rewiring and regeneration of damaged synaptic connections [72].

#### Stimuli

**Fig. 1:**
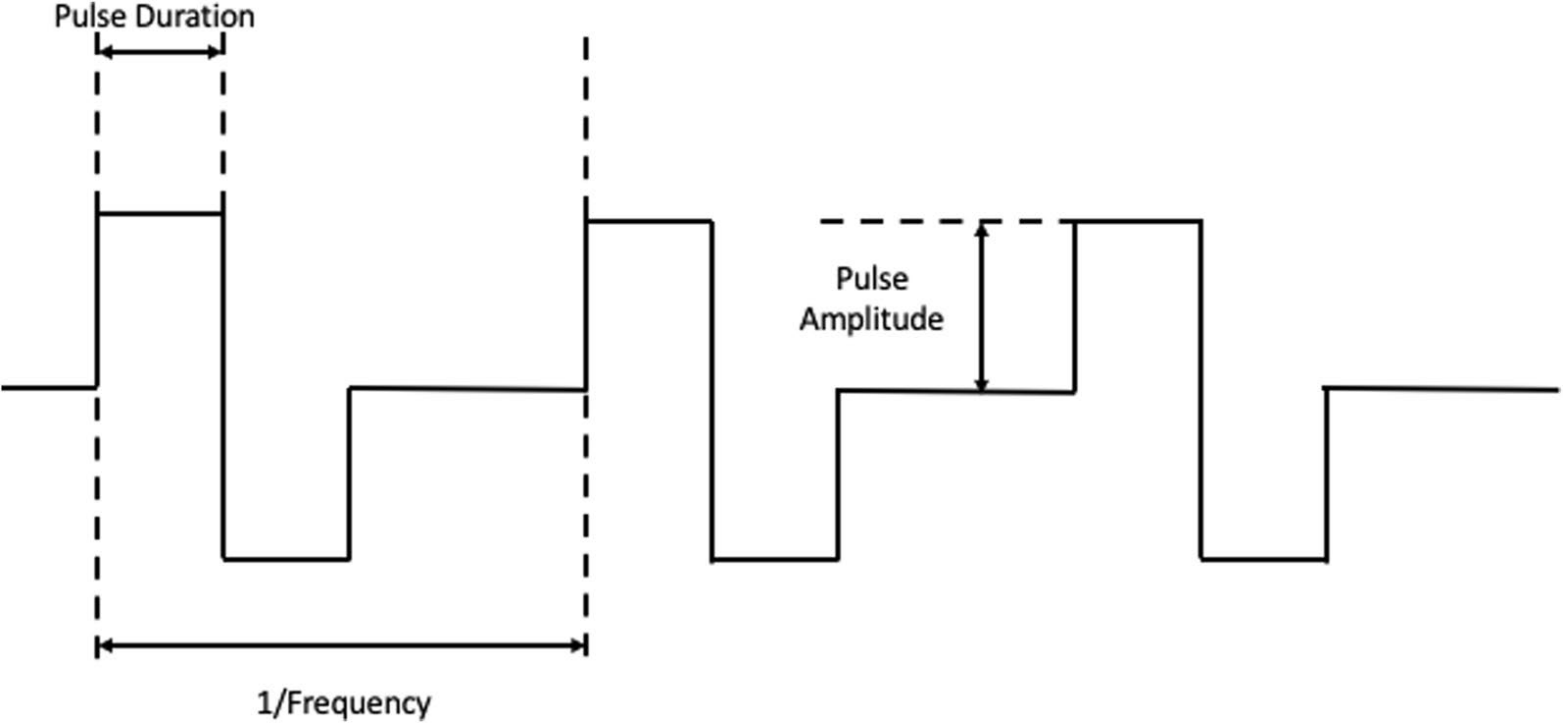
Functional electrical stimulation parameters: pulse duration, pulse amplitude, and pulse frequency

FES uses surface or implantable electrodes to deliver electrical stimuli. The placement of the electrode determines the selection of muscles stimulated and resulting movements. However, the optimal location of electrode placement and intensity of electrical stimuli requires trial and error to isolate the desired movement. The intensity of the electrical stimuli is determined by adjusting the duration and amplitude of the pulse (Fig. 1). Pulse duration is the time in which the stimulation is present, while pulse amplitude is the magnitude of the stimulation and determines which nerve fibers respond to the stimulation. As the intensity of the pulse increases, in either amplitude or duration, the current spreads and activates a larger cross-sectional area of skeletal muscle increasing the force exerted. Large nerves, which innervate large motor units, have the lowest threshold for stimulation and are recruited first, followed by small neurons and motor units. This phenomena is known as reverse recruitment and is the opposite of the physiologic size principle of motor neuron activation [73]. Unfortunately, this early recruitment of large muscles commonly leads to muscle fatigue, which may be mitigated to some degree through the modification of pulse frequency. Pulse frequency is the rate at which stimulation pulses are delivered. By increasing the pulse frequency, individual muscle twitches compound into a sustained contraction to produce movement called tetanic contraction. A minimum frequency of 16–20 Hz is required to induce contractions [74]. Higher frequencies create stronger contractions but also exacerbate muscle fatigue. Thus, a range of 20–50 Hz are typically used in FES.

**Fig. 2:**
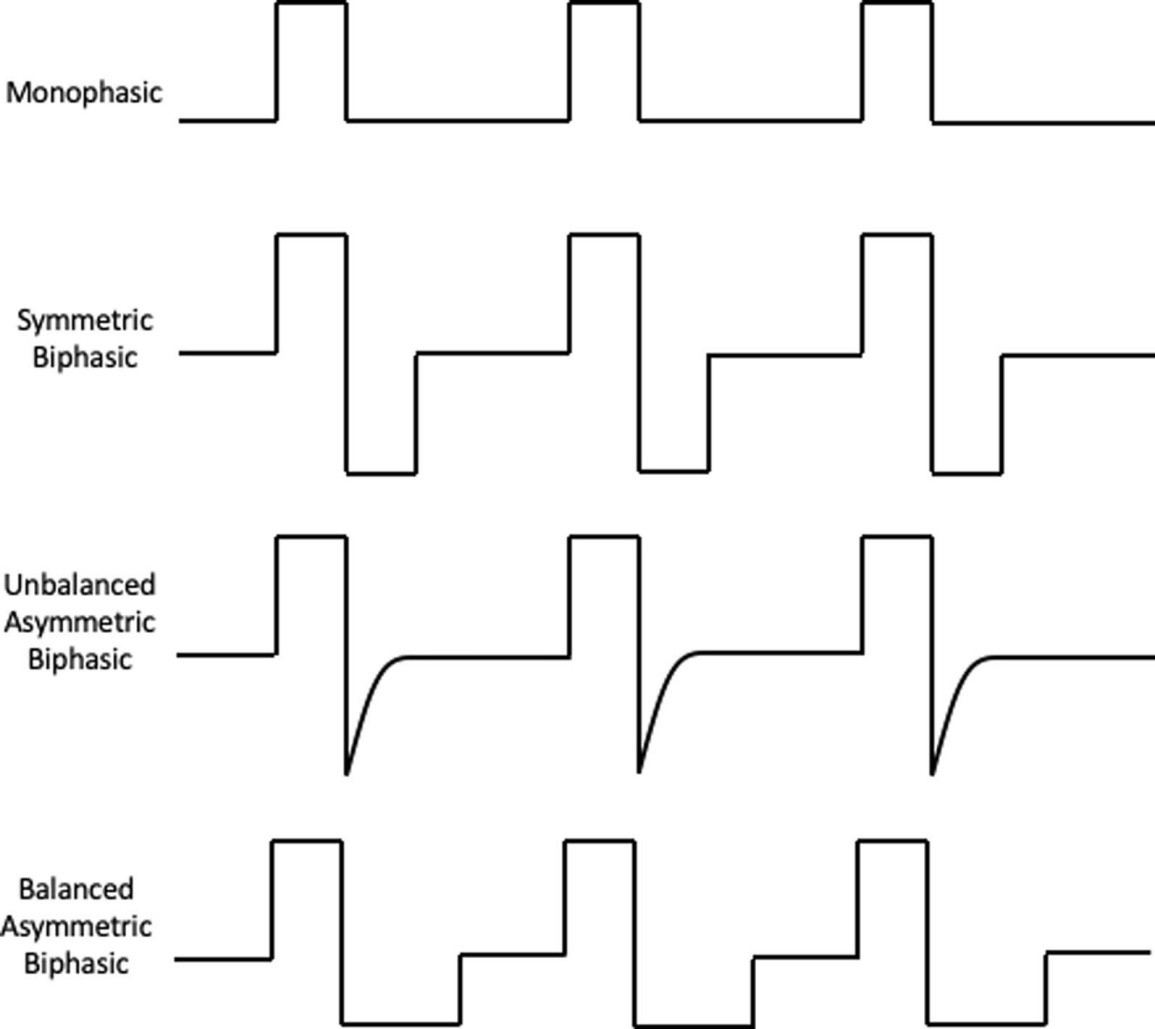
Pulse shapes for functional electrical stimulation

Pulses used in electrostimulation can be either monophasic, also known as direct current, or biphasic, also known as alternating current (Fig. 2). Monophasic pulses consist of a unidirectional pulse, whereas biphasic pulses are bidirectional with a positive and negative phase. The primary risk of monophasic pulses is thermal injury to surrounding tissue. Biphasic pulses can alternate anode and cathode electrodes (alternating biphasic pulses), which is believed to be safer for surrounding tissue. Biphasic pulses have a net charge of zero as the initial phase elicits an action potential in nearby nerves and the second phase balances the charge injection to protect surrounding tissue.

**Fig. 3:**
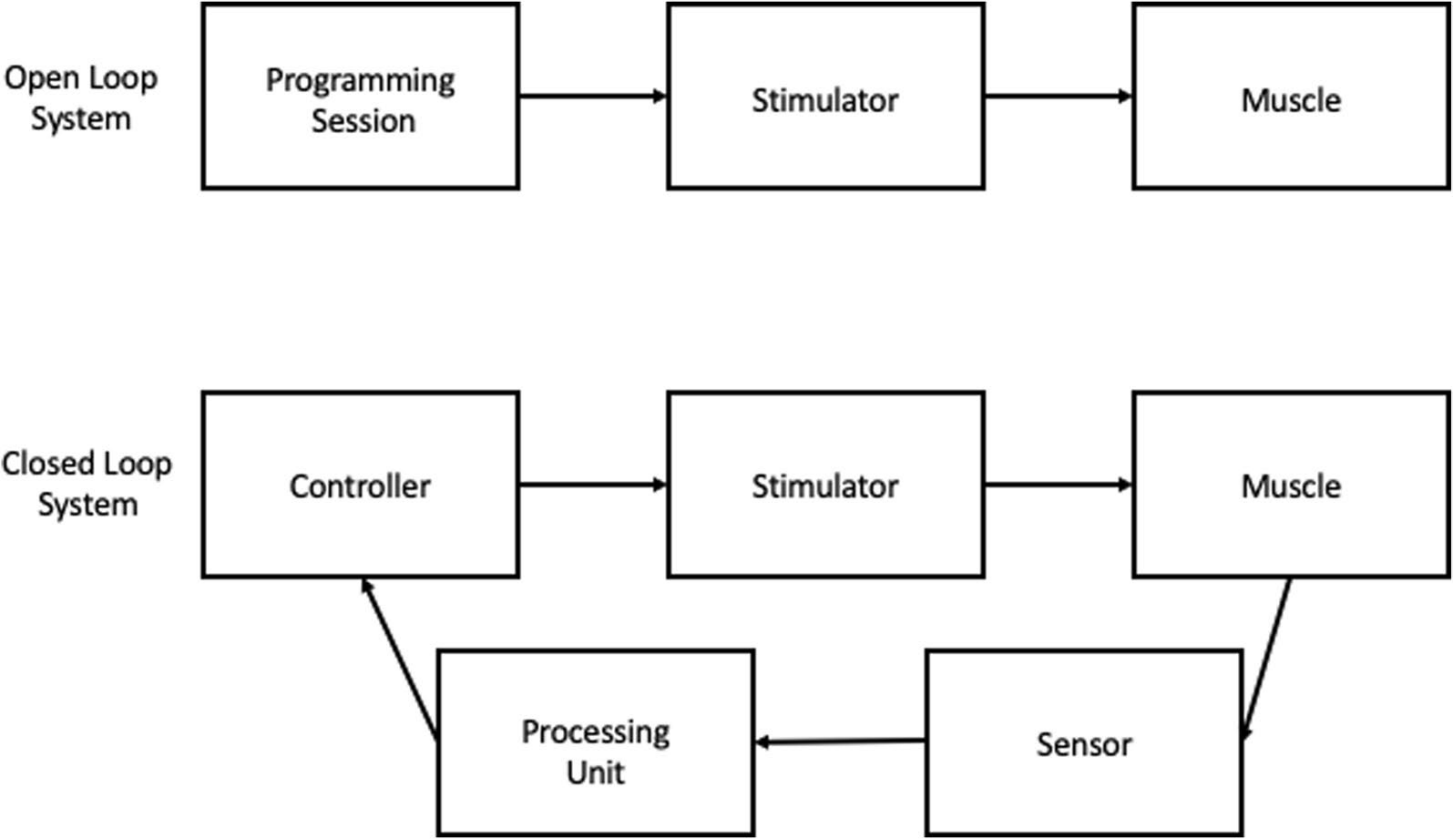
Closed and open loop systems. Open loop systems do not provide feedback. Closed loops systems have a feedback loop that continuously sends information back to the controller to self-correct

FES is either controlled as an open or closed loop system (Fig. 3). Open loop systems apply electrical current using fixed settings and do not incorporate biofeedback, and therefore lack the ability to self-correct. Alternatively, closed loop systems continuously relay contraction force and joint position information via sensors back to a computer to modulate input [75].

#### Therapeutic use

FES plays a prominent role in rehabilitation following SCI, mainly to restore extremity function. FES has been shown to increase muscle power output and resistance [76]. Multiple studies have validated the use of FES in helping to restore upper extremity function following SCI [77–80]. FES has also been used in rehabilitation of the lower extremity to improve gait parameters such as foot pulling acceleration, swing power, and ground impact force, ultimately resulting in improved walking speeds and more efficient system of muscle strength for gait [81–85]. A study using ankle weights to measure improvement in muscle strength after FES treatment for AIS A–C patients unable to stand demonstrated an average of 2–4× increase in power output in a 12 week study [76]. Additionally, FES has also been shown to improve patient transitions, spasticity, cardiovascular function, and pain [86–89]. Lastly, recent evidence has found that electrical stimulation increases the success rate of spinal fusion [90]. As such, functional or direct electrical stimulation could potentially be used to reverse and counter the bone loss and risk of fragility fracture in SCI patients.

#### Neuroprosthesis

FES technology has been integrated into neuroprostheses to control paralyzed muscles and improve functional independence. These systems comprise four major parts: the electrical stimulator, electrodes, sensors, and an orthosis. The electrical stimulator generates the electrical discharges that produce muscle contractions. These stimulators contain multiple channels, with each channel consisting of a pair (anode and cathode) of electrodes. Multiple channels are used to stimulate multiple muscles individually to produce functional movement. Electrodes are the interface between the external circuitry and the tissue and can be transcutaneous or implantable. Transcutaneous systems are noninvasive, do not require surgery, and are easy to reposition [74]. They can be connected to an external stimulator worn around the body that regulates and initiates the electrical stimulation, however, these systems are not suitable for stimulation of deep muscles and do not consistently achieve effective stimulations [73]. Implanted electrodes are surgically placed in the body, which allows for precise placement and direct stimulation of the desired muscles, resulting in repeatable and well-controlled contractions [73]. While reliable and effective, they have higher risks of complications, such as infections, due to their invasive nature. Additionally, their placement cannot be modified without additional surgery [51, 91, 92]. Sensors provide the biofeedback for the neuroprosthesis to achieve maximum functionality. Feedback-based control systems monitor the musculoskeletal system to alter the stimulation parameters in real time to achieve the desired movement. Finally, an orthosis provides additional structural assistance to perform desired movement by preventing muscle fatigue and helping patients conserve energy [93].

Hand and arm control are the most desired functions for patients with cervical SCI. FES neuroprosthesis have been developed to facilitate upper limb functions of reaching and grasping. The Freehand system developed by Hunter Peckham, Ron Triolo, and colleagues at the Cleveland FES Center was the first hand system to be granted United States Food and Drug Administration (FDA) approval [94]. The system consisted of implantable electrodes and a joystick to control the device. While it is no longer commercially available, a newer version, referred to as the implanted stimulator-telemeter (IST-12), was developed by the same team and has shown promising results in a clinical trial for improving the ability to grasp and manipulate objects [95].

After SCI, atrophied trunk musculature often lacks the required forces to control posture [96, 97]. Continuous FES can be used to stiffen trunk and hip extensors to stabilize the torso, correct kyphotic posture, improve ventilation, and normalize lateral vertebral alignment [98, 99]. Implanted electrodes at L1–L2 recruit lumbar erector muscles in combination with electrodes that activate the gluteus maximus improve trunk and hip extension. By activating these muscles, patients experience improved seated stability, seated posture, and enhanced bimanual reach [98]. However, this improvement cannot be maintained without constant stimulation. Further research is underway in a clinical trial investigating a trunk neuroprosthesis that is surgically implanted to facilitate trunk stability while sitting [100].

Neuroprostheses have shown promise in restoring the ability to stand and walk. FES used in combination with an ankle foot orthosis to provide support has helped patients activate the muscles in the lower extremity necessary for standing and walking [93]. Patients with implanted neuroprosthesis electrodes that continuously activate the erector spinae and gluteus maximus muscles for trunk and hip extension have been shown to maintain standing for greater than 10 minutes [101]. This small time frame enables patients to access wheelchair inaccessible areas and to utilize their upper extremities for activities other than maintaining balance with assistive devices. Furthermore, neuroprostheses have successfully reduced the torque and power output needed to initiate walking movement [102]. One of the most successful neuroprostheses for walking is Parastep. Parastep is an FDA approved device that uses transcutaneous electrodes over the peroneal nerves to allow ambulation [103]. Lower extremity neuroprostheses still face significant limitations due to the rapid onset of muscle fatigue and upper-body effort required to maintain balance with an ambulatory assistive device. A clinical trial is currently underway to investigate a new standing neuroprosthesis that uses multiple contact peripheral electrodes to slow fatigue onset and increase standing duration [104].

### Phrenic nerve pacing

High cervical spinal injuries carry the risk of altering respiratory function, which can result in respiratory failure. An alternative to ventilator dependence is diaphragmatic pacing via electrical stimulation of the phrenic nerve [105]. Phrenic nerve pacing has been used successfully for over 30 years, and a variety of implanted systems have been developed and commercialized [106]. Phrenic pacing has been shown to reduce time on the ventilator and may provide a full-time alternative to a ventilator [107]. Phrenic nerve pacing requires intact nerve function. However, pacing was recently achieved in patients with high cervical SCI (C3–5) and loss of phrenic nerve function via intercostal nerve grafting and implantable electrodes [108].

### Spinal cord stimulation

Spinal cord stimulation (SCS) is a neuromodulation technique used to treat neuropathic pain by virtue of its purported effect of blocking the transmission of pain signals through nociceptive nerve fibers entering the dorsal horn, similar to TENS [109]. SCS involves transcutaneous electrodes placed on the skin over the vertebral column or implanted epidural electrodes in the dorsal spinal cord. Increasing evidence shows that SCS also improves motor function via neuroplasticity following SCI [60, 110]. Recently, transcutaneous SCS has been shown to increase upper and lower extremity function, comparable to implanted SCS [111–117]. These findings are controversial and the rationale for the current Up-LIFT clinical trial that seeks to evaluate the effectiveness of noninvasive electrical SCS in treating upper extremity functional deficits in patients with chronic tetraplegia [118]. The advantages of transcutaneous SCS include its noninvasive application, affordability, and compatibility with conventional and commercially available stimulation devices. Recent advances in SCS include the delivery of spatiotemporal stimulation based upon gait kinematics and locomotor performance. However, this technology will require implantation for the foreseeable future to target specific areas of the spinal cord and stimulate unique muscles with precise timing. Implanted devices that can apply complex spatiotemporal patterns have reproduced voluntary control of locomotion in severely paralyzed patients [119–121]. Clinical trials are underway evaluating SCS in recovering lower extremity, bladder, bowel, and sexual function [122, 123]. Additionally, both transcutaneous and implanted SCS have been shown to improve autonomic cardiovascular dysfunction that occurs after SCI [124].

### Autonomic neuromodulation

SCI disrupts sympathetic vasomotor control, resulting in severe cardiovascular dysfunction. While pharmacological treatment of autonomic nervous system (ANS) regulation has demonstrated limited effectiveness, device-based neuromodulation has been shown to successfully modulate the cardiac ANS [125, 126]. Vagal nerve stimulation (VNS) has been shown to promote synaptic plasticity and improve autonomic instability and motor control in preclinical models with the potential to treat dysautonomia related to SCI [127–130].

An alternative method of autonomic neuromodulation is translingual neurostimulation. Translingual neurostimulation is a noninvasive method used to elicit neural changes in cranial nerves by targeting the anterior portion of the tongue activating the lingual branch of the trigeminal nerve [131, 132]. Prior research has shown that this method induces changes in the brainstem and cerebellum, specifically targeting areas important for postural drive [132–135]. As such, studies have shown improvement in balance and gait function in patients following SCI, stroke, and traumatic brain injury (TBI) [136–138].

### Sacral nerve stimulation

Sacral nerve stimulation (SNS) has been established as a treatment for urinary retention, frequency, and incontinence [139, 140]. Sacral nerve stimulation restores normal bladder function by facilitating storage and voiding and suppressing reflex bladder activity through adaptive neuronal plasticity [141]. SNS has successfully treated neurogenic bladder dysfunction via implanted sacral and transcutaneous sacral root, posterior tibial, and dorsal genital nerve stimulation [142–145].

### Galvanic vestibular stimulation

Galvanic vestibular stimulation (GVS) applies current at the mastoid process and activates afferent fibers of the vestibular nerve. Vestibulospinal neurons converge on spinal interneurons, promoting inhibitory or excitatory actions. These actions affect the tone of postural muscles, where stimulation of the anode results in hypotonia and stimulation of the cathode results in hypertonia. GVS can modulate the vestibulospinal tract and has been used to supplement the neurological examination by diagnosing and quantifying vestibulospinal deficits in patients with incomplete SCI [146]. GVS has been shown to reduce spasticity in SCI patients, and increase postural stability [147, 148].

### Transcranial direct current stimulation

Transcranial direct current stimulation (tDCS) is a noninvasive approach that delivers low-intensity direct current via electrodes placed on the head. tDCS is hypothesized to promote neuronal plasticity by altering membrane potential and cortical excitability [149]. Whether tDCS is depolarizing or hyperpolarizing, and inhibitory or excitatory, depends on the exact spatial locations of the contacts, the current path, the underlying geometry of the brain, and the degree of shunting to the scalp and skull. tDCS has been combined with motor training to promote activity-dependent neuronal plasticity, and has been shown to improve manual dexterity [150–152]. However, improvements in lower extremity motor function remain controversial [153]. A clinical trial is currently underway to investigate transcutaneous direct current stimulation (tcDCS) on walking function in individuals with incomplete SCI [154]. Variations of tDCS include alternating current stimulation (ACS) and oscillating field stimulation (OSF). ACS delivers transcranial alternating current electrical stimulation and has been shown to decrease pain perception and increase muscle work in SCI patients [155]. OSF has shown promise for remyelination and axon regeneration in preclinical models, but failed to significantly improve motor status in humans [156–158].

### Future directions

**Fig. 4:**
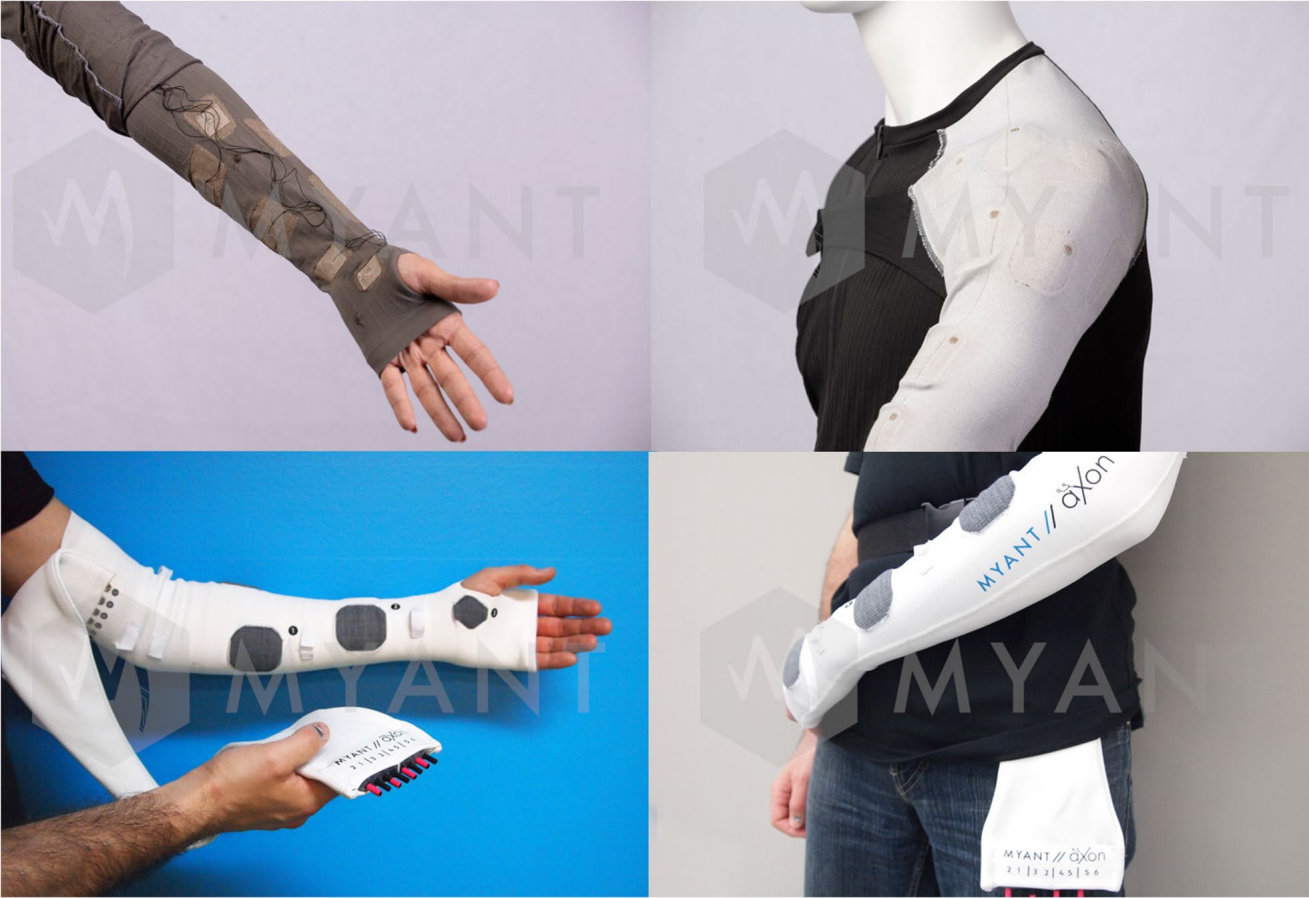
Myant wearable garments. Garments can be worn under clothes and provide stimulation through electrodes embedded into clothing. Permission to print photos granted by Myant (Toronto, Canada)

Current trends in health care delivery have encouraged research in methods to deliver FES on an outpatient basis. One solution is a garment-based stimulation technology developed by the textile computing company Myant (Toronto, Canada) (Fig. 4). These garments combine cloth with silver thread or conductive layers to embed electrodes into the clothing to deliver electrical stimulation. These wearable garments should be customizable, cost effective, versatile, and durable compared with alternative options, and allow for independent application by the patient [159].

Previously, TENS/FES/SCS has been used to restore function using various command interfaces including electromyography (EMG), voice recognition, mouth sticks, chin-controlled joysticks, sip-and-puff, inertial measurement units (accelerometer, gyroscope), eye gaze, head tracking, and tongue movement [160–167]. Brain–computer interfaces (BCI) are an emerging technology with the potential to be used in SCI rehabilitation. Electroencephalogram (EEG)-based BCI has been shown to translate a task-related motor intention neural signal into a specific command [168]. Recently, progress has been made in using intracortical brain–computer interfaces to interpret intended movement signals and command transcutaneous and intramuscularly implanted FES electrodes to generate the intended movements of reaching and grasping [169–173]. Furthermore, clinical trials are underway investigating a spine interface that will bridge spinal cord lesions by interpreting neural information above a lesion and transmitting it to electrodes below the lesion [174].

### Neuromodulation for SCI

The direction of care for patients with SCI can often be complex given the numerous modalities available to assist in rehabilitation. Spinal surgeons may be inclined to place any number of implantable devices during the index spinal cord stabilization surgery. Unfortunately, due to the nature of SCI, the full extent of a patient’s functional limitations may not be known until significant time has passed. As such, this poses a challenge for determining the proper intervention early in the disease course when early intervention could drastically improve long-term functional recovery.

Preoperatively, spine surgeons must consider the extent of SCI using objective scales such as the AIS, as well as their own clinical judgement regarding the long-term recovery of the patient. In the future, surgeons may utilize advanced diffusion tactography sequences on magnetic resonance imaging (MRI), which have been shown to detect functional changes in SCI patients to help predict outcomes and guide treatment [175]. Although the entire course of a SCI is difficult to predict at onset, certain extremes of injury can be predicted based on initial examination findings. These dysfunctional injuries may benefit from early intervention in high probability areas of functional impairment. For example, a patient with complete cord transection resulting from high cervical spinal injury will almost surely be placed on a ventilator for immediate life-saving support and, therefore, have a high likelihood of showing early improvement with a phrenic nerve pacer to prevent atrophy of respiratory musculature. The implication of such interventions would be the prevention of multiple surgeries when anticipated sequelae could be prevented during the index procedure.

Early neurorehabilitation with electrical stimulation has the potential to reduce morbidity and mortality in patients with SCI. Future research should focus on ways to organize and plan early management to prevent unnecessary surgeries, while increasing functionality and recovery. Additionally, further consideration is required to compare the risks and benefits of these interventions as technology continues to flourish in the wake of faster, more precise, and effective techniques.

## Conclusion

Electrical stimulation can be used in various forms to improve the well-being and functionality of patients with SCI. The scope of electrical stimulation continues to grow as more advanced technologies and interventions are developed and studied. The neuroplasticity induced by electrical stimulation portends a promising future for developing better therapeutic interventions that can lessen the functional disability and enhance the quality of life of patients with SCI. The prevalence of electrical stimulation will likely increase in the future, with neuroprosthetic devices playing an important role in rehabilitation.

## Data Availability

Not applicable.

## Abbreviations

SCI: Spinal cord injury
CAD: Coronary artery disease
UTI: Urinary tract infection
TENS: Transcutaneous electrical neural stimulation
NEMS: Neuromuscular electrical stimulation
SCS: Spinal cord stimulation
ANS: Autonomic nervous system
VNS: Vagal nerve stimulation
SNS: Sacral nerve stimulation
GVS: Galvanic vestibular stimulation
tDCS: Transcranial direct current stimulation
tcDCS: Transcutaneous direct current stimulation
ACS: Alternating current stimulation
OSF: Oscillating field stimulation
BCI: Brain–computer interfaces
EEG: Electroencephalogram
